# Tricho-dento-osseous syndrome and precocious eruption

**DOI:** 10.4317/jced.53348

**Published:** 2017-03-01

**Authors:** Parul Jain, Rahul Kaul, Subrata Saha, Subir Sarkar

**Affiliations:** 1 Post Graduate Student, Department of Pedodontics and Preventive Dentistry, Dr. R. Ahmed Dental College and Hospital, Kolkata, West Bengal, India; 2 Professor, Department of Pedodontics and Preventive Dentistry, Dr. R. Ahmed Dental College and Hospital, Kolkata, West Bengal, India; 3 Professor & HOD, Department of Pedodontics and Preventive Dentistry, Dr. R. Ahmed Dental College and Hospital, Kolkata, West Bengal, India

## Abstract

Tricho-dento-osseous syndrome (TDO), an uncommon form of ectodermal dysplasia is an autosomal dominant genetic disorder which is characterized by inherited defects in tissues arising from epithelial-mesenchymal interaction. Genetic studies have revealed that it is caused by mutation in the DLX3 gene. TDO presents with a great phenotypic heterogeneity and studies have suggested that this heterogeneity is the result of environmental factors or other genetic modifiers. In this article, we report a case of TDO in which the child had typical clinical features of hair, teeth and bone defects, as seen in TDO. Parents of the child were unaffected. Genetic analysis of the child revealed mutation in DLX3 gene. The child also showed precocious eruption of the permanent molars, a clinical feature which has been rarely reported. We suggest that the precocious eruption seen in TDO is probably due to a markedly increased osteoblastic activity.

** Key words:**Tricho-dento-osseous syndrome, DLX3 gene, precocious eruption.

## Introduction

Ectodermal dysplasias (EDs) comprise a large, heterogeneous group of inherited disorders that are defined by primary defects in the development of two or more tissues derived from embryonic ectoderm. The tricho-dento-osseous syndrome (TDO), OMIM #190320, is an uncommon form of ectodermal dysplasia. It is an autosomal dominant genetic disorder which is characterized by inherited defects in tissues arising from epithelial-mesenchymal interaction; primarily the hair, teeth and bones from which the condition derives its name. The term “tricho-dento-osseous” (TDO) was first coined by Lichtenstein *et al.* in 1972 (1,2), although the first case was reported in 1966 by Robinson and co-workers (3). Genetic analysis in most studies has shown a mutation in the DLX3 gene (4).

Till date, literature reports three large kindreds and related families in the United States (Virginia, North Carolina, and Tennessee) (1,4,5) to be affected with TDO. In addition to these well documented kindreds, there have been isolated case reports of TDO from all around the world (6). TDO presents with a great phenotypic heterogeneity which can make the diagnosis somewhat confusing (7). The distinct, severe dental defects are the most reliable of all signs in TDO. Differentiating TDO from other types of dental defects such as hypomaturation hypoplastic amelogenesis imperfecta with taurodontism (AIHHT), both of which show overlapping clinical features, is necessary so as to lead to discoveries about causes and bring about advances in treatment, which will ultimately lift all our collective hopes for the future.

The purpose of this article is to report an isolated case of tricho-dento-osseous syndrome in a child who had typical clinical features of the teeth, hair and bone defects, as seen in TDO. Genetic analysis of the child revealed a mutation in DLX3 gene, which is probably sporadic, as the parents of the child were not affected with TDO. Besides the typical clinical features, the child also showed precocious eruption of the permanent molars; a feature which has been rarely reported. We suggest that the precocious eruption seen in TDO is probably due to a markedly increased osteoblastic activity.

## Case Report

A 4 year old male child reported to our Department of Pedodontics and Preventive Dentistry with the chief complaint of discoloured teeth. Medical history was non-contributory. His mother gave a history of an uncomplicated, full-term pregnancy resulting from a nonconsanguineous marriage.

On general examination, the child appeared well nourished. His measured height was 96.25cm. Parents reported normal mental development of the child. He had sparse, discoloured hair and had a history of 3-4 hair cuts. The child had a broad forehead which appeared prominent on lateral view. The measured head circumference was 51.25cm, which falls in the 50th percentile. There was no evident splitting of the superficial layer of finger nails or toe nails.

**Figure 1:**
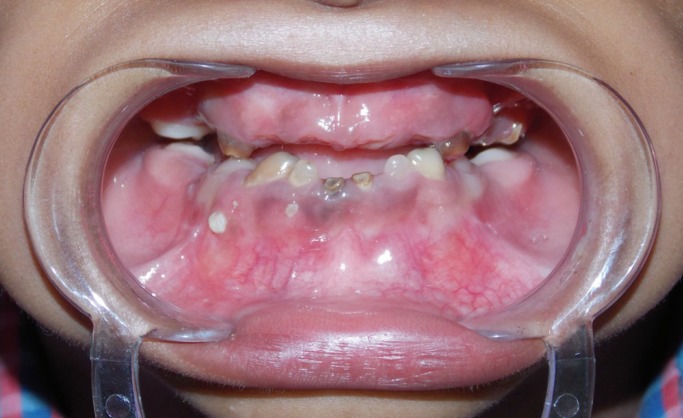
Intra-oral view showing severly attrited teeth, visible pulp chambers and class III anterior occlusal relationship.

Dental examination (Fig. 1) revealed eruption of all four permanent first molars. Primary teeth had a history of extraction due to formation of dental abscess. The remaining primary teeth were opaque and yellowish-brown in colour. Severe attrition of mandibular primary incisors and maxillary primary canines were seen. Pulp chambers of maxillary and mandibular primary canines were evident through the thin enamel layer. Both posterior and anterior occlusal relationships revealed a Class III tendency.

**Figure 2:**
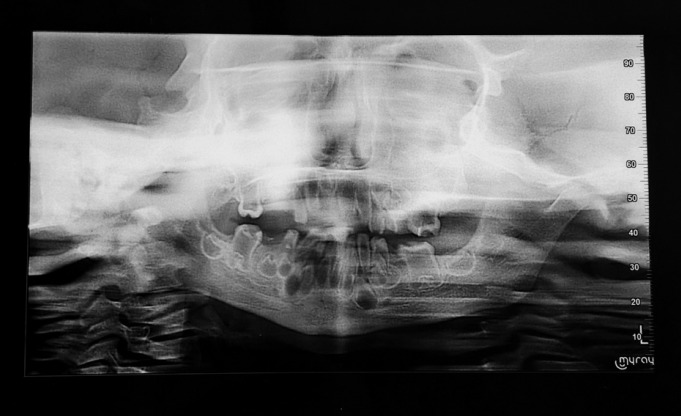
Panoramic radiograph showing taurodontic teeth and thickened cortical bone.

Panoramic radiograph (Fig. 2) of the child showed increased sclerosis of the mandibular cortical bone. All the remaining primary teeth and the erupted first permanent molars appeared taurodontic. The enamel appeared extremely thin, the pulp chambers were enlarged with greater apico-occlusal height and the furcation was displaced apically. Radiographic contrast between enamel and dentin was reduced. As root formation of the first permanent molars was not complete, an objective assessment of taurodontism was not done.

**Figure 3:**
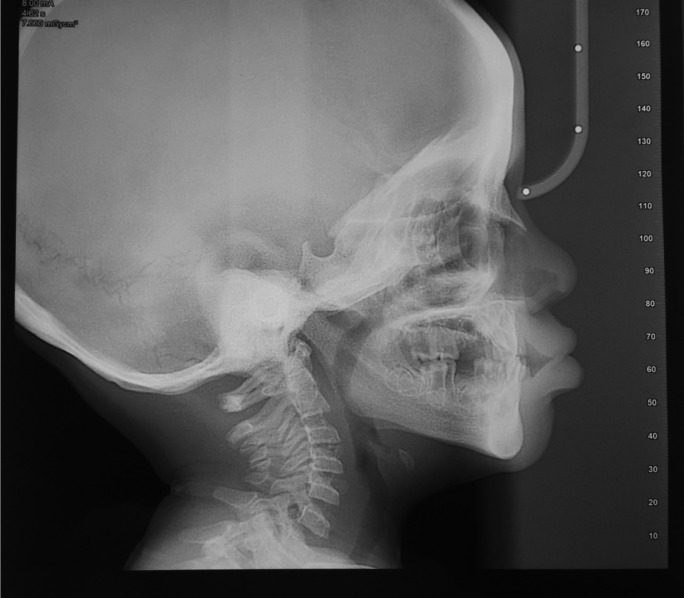
Lateral cephalogram showing increased density of mandible and base of skull, reduced frontal sinus pneumatization and class III skeletal relationship.

Lateral cephalogram (Fig. 3) examination showed increased density of the mandible (especially the symphysis region) and the base of the skull. Frontal sinus pneumatization was not very apparent. Cephalometric analysis revealed a Class III skeletal pattern because of a retrusive maxilla (reduced SNA).

Genetic analysis reports of the child revealed a 4-bp mutation in the DLX3 gene.

The child’s parents had no abnormal hair, bone or dental findings and were apparently unaffected by the condition. Parents were made aware about the dental defects of the child and the associated condition. Counselling was done and they were explained about the need for regular follow ups. Extraction of symptomatic primary teeth was done. Stainless steel crowns were cemented on all four erupted permanent molars with glass ionomer cements after minimal crown preparation. Split acrylic removable denture was given for the maxillary arch. The patient provided their written informed consent.

## Discussion

Tricho-dento-osseous (TDO) syndrome is a highly penetrant, autosomal dominant genetic disorder which is characterized by inherited defects in tissues which arise from epithelial-mesenchymal interaction. As per studies (6), the minimal diagnostic criteria for TDO should include enamel hypoplasia, posterior teeth with taurodontism, autosomal dominant inheritance pattern, tightly curly hair at birth and/or radiographic evidence of bone sclerosis.

As per early epidemiologic investigations, Shapiro (7) suggested classification of TDO into three subtypes, TDO - I, II and III, based on clinical and radiographical features. However, further studies (4) largely discounted this classification on genetic grounds and it has been suggested that TDO is a condition with great phenotypic variation. Studies have also shown that TDO and AIHHT are genetically distinct conditions (6).

TDO syndrome is a genetic disorder arising because of a mutation in the DLX3 gene. This gene is a member of the DLX family (related to Drosophila distal-less gene, which is involved in head and limb development). DLX3 is located in a tail-to-tail configuration with another member of the gene family (DLX7) on the long arm of chromosome 17, and encodes a homeodomain protein. Early DLX3 expression is localized to first and second branchial arches that will give rise to odontoblast and craniofacial structures. In the later stages of development, it is expressed in tissues arising from epithelial-mesenchymal interaction such as skin, hair follicles, otic and olfactory placodes, limb buds, placenta and tooth germs. Their main action in these tissues is to induce cellular differentiation by regulating gene expression through their DNA-recognition properties (8). For these ectodermally derived organs, DLX3 gene and p63 gene (master regulatory gene of multi-layered epithelia) act as a part of a series of regulatory cascades of differentiation. Degradation of DNp63a, a specific product of the p63 gene, is induced by DLX3 expression (9).

Regarding the effect of DLX3 on mineralized tissue, its influence varies specifically according to the terminal differentiation for each cell type. Direct involvement of DLX3 has been shown in osteoprogenitor cells, where it promotes the expression of bone matrix proteins such as type 1 collagen, bone sialoprotein, osteocalcin, and alkaline phosphatase. The expression of osteocalcin and RUNX2 is actually because of a combined action of DLX3, with DLX5 and MSX2 (10,11). Role of DLX3 has also been shown in osteoclastogenesis. Zhao *et al.* (10) investigated the effect of DLX3 on osteoclastogenesis and concluded that DLX3 is involved in the process of osteoclastic differentiation through microRNA-124 and when compared to wild type DLX3, mutant DLX3 mutation had a stronger suppression on osteoclastogenesis.

An increased DLX3 expression has also been shown during the osteogenic differentiation of neural crest derived stem cells of dental follicle (DFCs). Additional osteogenic transcription factors such as MSX2, RUNX2, DLX5, or OSTERIX which are seen in osteoprogenitor cells, are not involved in osteogenic differentiation of DFCs, suggesting an important role of DLX3 in the progression of differentiation of these cells (8).

During tooth development, DLX3 has also been found to up-regulate the enamel matrix protein genes Amelx, Enam, Odam, and Klk4 (12).

Mutant- DLX3 (responsible for TDO), disrupts all these regulatory functions. Disruption of odontoblastic cytodifferentiation, which leads to odontoblast apoptosis has been shown in transgenic mice with mutant DLX3 (8). However, TDO patients do not exhibit overt skin phenotype. Costanzo *et al.* (9) suggested that co-expression of wild and mutant DLX3 in skin effectively regulates DNp63a protein level, thereby protecting patients from developing severe p63 associated skin abnormalities.

To date, six DLX3 mutations are known to exist. In general, missense mutations, which change conserved amino acids in the homeodomain (129-188 amino acids), result in the most severe clinical phenotypes. Mayer *et al.* (13) reported a case with I175T mutation. Nieniman *et al.* (14) reported R133P and S182F mutation in two families of Finnish origin. Price *et al.* (4) found that the TDO phenotype in all the six North Carolina families and the Virginia family was associated with a 4-bp deletion in the DLX3 homeobox. They suggested that these families perhaps inherited this deletion mutation from a common ancestor. They also suggested that the clinical heterogeneity was not genetic in nature, but was the result of environmental factors and/or other genetic modifiers. Similarly, the 2-bp deletional mutation (15) has been identified in multiple families with different ethnicities and with different phenotypic expressions.

In the present case, the child was probably a case of sporadic mutation of DLX3 gene as the parents of the child were unaffected by the condition. Besides the hair, teeth and bone defects, the child’s clinical and radiographic examination revealed frontal bossing, a class III skeletal pattern because of maxillary retrusion and precocious eruption of the permanent first molars.

Tooth eruption results from an interplay between osteoblastic and osteoclastic activity. A continuous remodelling process takes place around an erupting tooth. In the present case, the child had a normal dental age but had a markedly increased osteoblastic activity, as was evident by the significantly increased density of the mandible and the skull bones. A great increase in osteoblastic activity is perhaps responsible for precocious eruption. Literature has mostly reported cases of TDO associated with delayed eruption. We hypothesize that in cases of TDO syndrome, which present with increased osteoblastic activity, there is a shift of equilibrium towards bone deposition all around the erupting tooth, and the consequence is delayed eruption. But in cases which have highly increased osteoblastic activity, the large amount of bone deposition around the root region of the erupting tooth will create sufficient pressure to disrupt the increased osteoblastic activity at the coronal end of the erupting tooth. This compressive mechanical force will stimulate osteoclast differentiation, resulting in precocious eruption.
